# Study of ethion and lipopolysaccharide interaction on lung in a mouse model

**DOI:** 10.1186/s42826-020-00055-z

**Published:** 2020-07-29

**Authors:** Geetika Verma, R. S. Sethi

**Affiliations:** College of Animal Biotechnology, Guru Angad Dev Veterinary and Animal Sciences University, Ludhiana, Punjab India

**Keywords:** Ethion, TLR-4, IL-1β, TNF- α, Genotoxicity, Organophosphates

## Abstract

Ethion is an organophosphate used commonly in India despite being banned in many other countries. The present study was designed to study the interaction of ethion and lipopolysaccharide (LPS) together on lung after single low dose ethion exposure. Mice (*n* = 20) were alienated into control and treatment groups (*n* = 10 each). The treatment group was orally fed ethion (8 mg/kg/animal/day) dissolved in corn oil. The animals (*n* = 5 each) from both the groups were challenged with 80 μg *Escherichia coli* lipopolysaccharide (LPS) intranasally and the remaining animals (*n* = 5 each) were administered normal saline solution after 24 h. Ethion along with LPS induced lung inflammation as indicated by increased neutrophils and total leukocyte count (TLC) in broncheoalveolar lavage fluid. Ethion induced histomorphological alterations in lung as shown by increased pulmonary inflammation score in histopathology. Real time PCR analysis showed that ethion followed by LPS resulted significant (*p* < 0.05) increase in pulmonary Toll-like receptor (TLR)-4 (48.53 fold), interleukin (IL)-1β (7.05 fold) and tumor necrosis factor (TNF)-α (5.74 fold) mRNA expression. LPS co-exposure suggested synergistic effect on TLR4 and TNF-α mRNA expression. Ethion alone or in combination with LPS resulted genotoxicity in blood cells as detected by comet assay. The data suggested single dietary ethion exposure alone or in conjunction with LPS causes lung inflammation and genotoxicity in blood cells.

## Introduction

Pesticides play a crucial role in mitigating the food demands of large growing population worldwide. The ban on the use of much persistent organochlorine pesticides increased the usages of less persistent but more toxic organophosphorus pesticides (OPs) [1]. Ethion, an OP, is regularly used over range of crops and is helpful in managing ectoparasites in veterinary practice [2]. However, several studies have reported high level of its residues in drinking water, vegetables [3] and human colostrum [4] suggesting entry of ethion into food chain. Further, the personnel engaged in the manufacturing plants and agriculture sector are at a major risk of ethion exposures. Ethion poisoning cases result mainly because of lack of awareness and knowledge regarding ethion hazards, inadequate use of personal protective equipment and compromised safety standards.

Short term exposures to high ethion concentrations are more dangerous and result clinical toxicity exhibited by abdominal pain, vomiting, diarrhoea, excessive secretions and respiratory distress followed by death [5]. Acute exposure to herbicide such as paraquat impact the lung health, pathological damages and increased toll-like receptor (TLR)-4, pulmonary tumor necrosis factor (TNF)-α, interleukin (IL)-1β and nuclear factor (NF)-κB p65 levels [6]. We have reported that long term dietary exposures to ethion and lipopolysaccharide (LPS) cause lung damage and genotoxicity [7]. It arouses our interest to evaluate the effects of ethion alone and along with LPS on lung after single low dose of ethion. Although incidences of acute ethion poisoning has been reported [4, 5] yet the pathogenesis of lung injury at molecular level following single ethion exposure has not been completely elucidated.

TLRs are the important constituent of the innate immune response and TLR4 is a pattern recognition receptor in lung injury [8]. TLR4 activates macrophages, neutrophils and other immune cells leading to production of various cytokines, chemokines and proinflammatory mediators like IL-1β and TNF-α [9]. IL-1β and TNF-α are the most important cytokines involved in the acute lung inflammation [10, 11] and hence may contribute to the lung’s responses to ethion-induced injury and inflammation.

Endotoxins are frequently prevalent in agricultural environment so there remains a strong possibility that farm workers may get co-exposed to pesticides and endotoxins [12]. Lipopolysaccharide (LPS), an endotoxin, is an important constituent of cell wall component in Gram negative bacteria that ligates TLR4 to initiate lung inflammation [9]. The studies regarding interaction of LPS and pesticide on cytokine expression [13]; apoptosis [14] and lung immunity [15] suggested synergistic effect of the combination of LPS with pesticide compared to pesticide alone. We have reported that LPS interacts with various classes of pesticides to alter the magnitude of pulmonary damage [2, 16–21] as well as genotoxicity [7, 22]. Thus, exposure to LPS may deteriorate the health of ethion exposed subjects by exaggerating the harmful effects of ethion. However, there are very limited data on the pulmonary and genotoxic effects following single dietary ethion exposure alone or in combination with LPS. Hence, we tested the hypothesis that single low dose dietary exposure to ethion alone or in conjunction with LPS cause lung inflammation and genotoxicity in a mouse model.

## Materials and methods

### Experimental animals

A total of twenty healthy Swiss albino male mice, aging 6–7 weeks, were maintained at small animal house facility of Guru Angad Dev Veterinary and Animal Sciences University (GADVASU), Ludhiana under the guidelines of the Committee for the Purpose of Control and Supervision of Experiments on Animals (CPCSEA), India. The experiment protocols were approved by the Institutional Animal Ethics Committee of the university (VMC/14/2413–43). Animals were acclimatized for 1 week before start of the experiment and were provided synthetic pelleted diet (Ashirwad Industries, Chandigarh) and water ad libitum.

### Dosages and exposure schedules

**Fig. 1:**
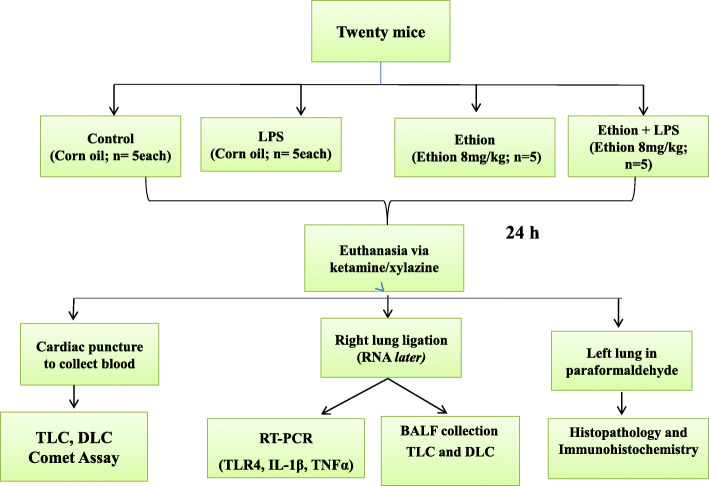
Schematic diagram depicting the layout of the experiment

The schematic representation of experimental design is given in Fig. 1. The animals (*n* = 20) were divided randomly into two groups: one treatment and one control (*n* = 10/group). The treatment and control group were orally fed 8 mg kg^− 1^ of ethion [Analytical grade, PESTANAL® (45477), Sigma, India] dissolved in corn oil (C8267, Sigma, India) and only corn oil, respectively for 24 h. The oral LD_50_ of ethion in mouse is 40 mg kg^− 1^ [23] and the lethal oral dose of ethion in humans is between 50 and 500 mg kg^− 1^ [24]. Therefore, the selected dose is much lower than concentration reported to show clinical symptoms. None of the animal died during the course of experiment.

After 24 h, five animals from each group were administered 80 μl *E. coli* LPS (L3129; Sigma, India; 1 mg/ml/animal) via intranasal route while the remaining five animals from each group were given 80 μl of normal saline solution (NSS) via same route. The animals were euthanized after 9 h of LPS/NSS exposure and whole blood, broncheoalveolar lavage (BAL) fluid and lungs were collected for further experimentation as mentioned earlier in our previous studies [7, 17].

### Total leukocyte count and differential leukocyte count analysis

Total leukocyte count (TLC) and differential leukocyte count (DLC) analysis were performed as per standard protocols of our lab [17]. Briefly, blood/BAL fluid (20 μl) and whole blood cell (WBC) diluting fluid (380 μl) were mixed and then cells were counted for TLC analysis. A blood/BAL fluid smear was prepared and stained with Leishman stain followed by counting of neutrophils and/or lymphocytes at x 40 for DLC analysis.

### Haematoxylin and eosin staining

The left lung was processed for sectioning (5 μm thick) followed by staining with haematoxylin and eosin to observe the histopathological changes using × 10 and × 40 objectives. Morphological changes in lungs were observed and graded semi-quantitatively (0, normal/absent; 1, mild; 2, moderate; 3, severe) for parameters like peribronchial infiltration, perivascular infiltration, sloughing of epithelium, thickening of alveolar septa and increase in perivascular space as described earlier [17]. The histopathological changes were expressed as pulmonary inflammation scores. The sample identity was not disclosed to the evaluator.

### Quantitative real-time PCR (qPCR)

The right lung was subjected to qPCR to detect TLR-4, IL-1β and TNF-α mRNA expression. Briefly, total RNA was isolated manually and reverse transcribed to cDNA followed by reaction mixture preparation using Quantifast SYBR® Green PCR kit (Qiagen, India). The reaction was performed in duplicate in RT-PCR (BioRad, USA) with β- actin as an endogenous control. The primer sequences for TLR-4, IL-1β and TNF-α were same as described earlier [7]. Each reaction included initial denaturation (94 °C for 1 min), denaturation (94 °C for 30 s), annealing (30 s) and extension (72 °C for 30 s) followed by a final extension (72 °C for 5 min). The number of PCR cycles was limited to 25–30. Data analysis was done by the ΔCT method for relative quantification.

### Immunohistochemistry

Immunohistochemistry was carried on the paraffin sections of the left lung as per standard protocol of our lab [25]. The sections were processed and incubated with primary antibodies against TLR-4 (sc12511; Santa Cruz; dilution 1:400), IL-1β (sc-1252, Santa Cruz; dilution 1:200) and TNF-α (sc1350; dilution 1:2000) for 1 hour followed by a suitable secondary antibody (Dako P0449; dilution 1:800) for 30 min. Color development was done with a commercial kit (SK4100; Vector Laboratories, USA) followed by counter staining with haematoxylin.

### Single cell gel electrophoresis (comet assay)

Briefly, blood (5 μL) and low melting point agarose (LMPA, 95 μL) were mixed and layered over normal melting agarose coated slides which were then subjected to electrophoresis and then viewed under a fluorescence microscope (Nikon Eclipse 90*i;* excitation:420–490 nm, barrier:520 nm) [9]. Fifty cells per sample were analyzed by Open Comet 1.3 [26].

### Statistical analysis

The data were subjected to one-way analysis of variance (ANOVA) followed by Tukey’s post-hoc test. Data presented as mean ± standard error (SE) considered statistically significant at *p* < 0.05. GraphPad Prism 6 software was employed for graphical representation and analysis of data. Each and every group was analysed and compared with each other.

## Results

### Total leukocyte count and differential leukocyte count analysis

#### Blood

**Fig. 2:**
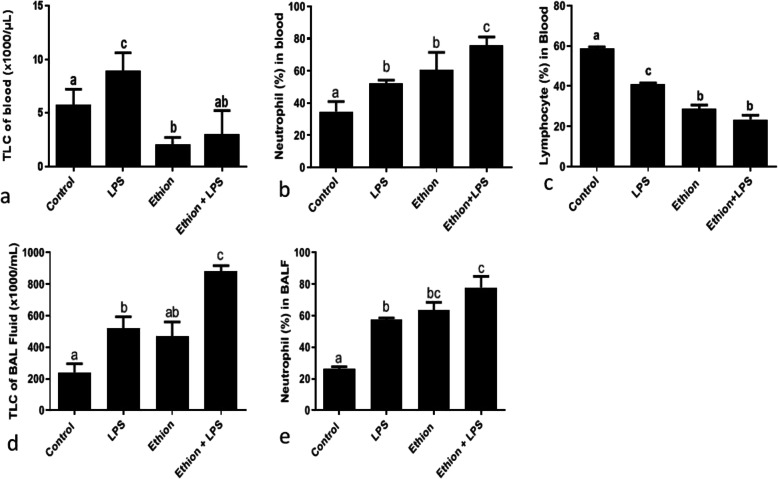
**a** TLC (× 10^3^/μl) of blood, **b** nNeutrophil, **c** lLymphocyte (%) in blood; **d** TLC (× 10^3^/ml) and **e** nNeutrophil (%) of BAL fluid after single dietary ethion exposure alone or in combination with LPS. **a,b,c,d**: no common superscript (**a, b, c, d**) between two levels of an effect indicates significant difference (*p* < 0.05) among groups

LPS exposure showed increase (*p* < 0.05) in TLC of blood compared to control (Fig. 2a). However, ethion exposure resulted decrease (*p* < 0.05) in TLC of blood. Further LPS or ethion resulted neutrophilia and lymphocytopenia (*p* < 0.05) compared to control (Fig. 2 b, c). Ethion in combination with LPS did not alter TLC and percentage of lymphocytes compared to individual ethion group (Fig. 2 a, c). However, the combination significantly increased the neutrophil percentage compared to individual ethion or LPS group (Fig. 2b).

#### Bronchoalveolar lavage fluid

LPS increased (*p* < 0.05) TLC of BAL fluid along with neutrophilia as compared to control (Fig. 2 d, e). Ethion did not alter TLC of BAL fluid but increased (*p* < 0.05) the neutrophil percentage. However, ethion in conjunction with LPS increased TLC (*p* < 0.05) compared to ethion or LPS group and resulted neutrophilia (*p* < 0.05) compared to control and LPS group (Fig. 2d, e).

### Lung histopathology

**Fig. 3:**
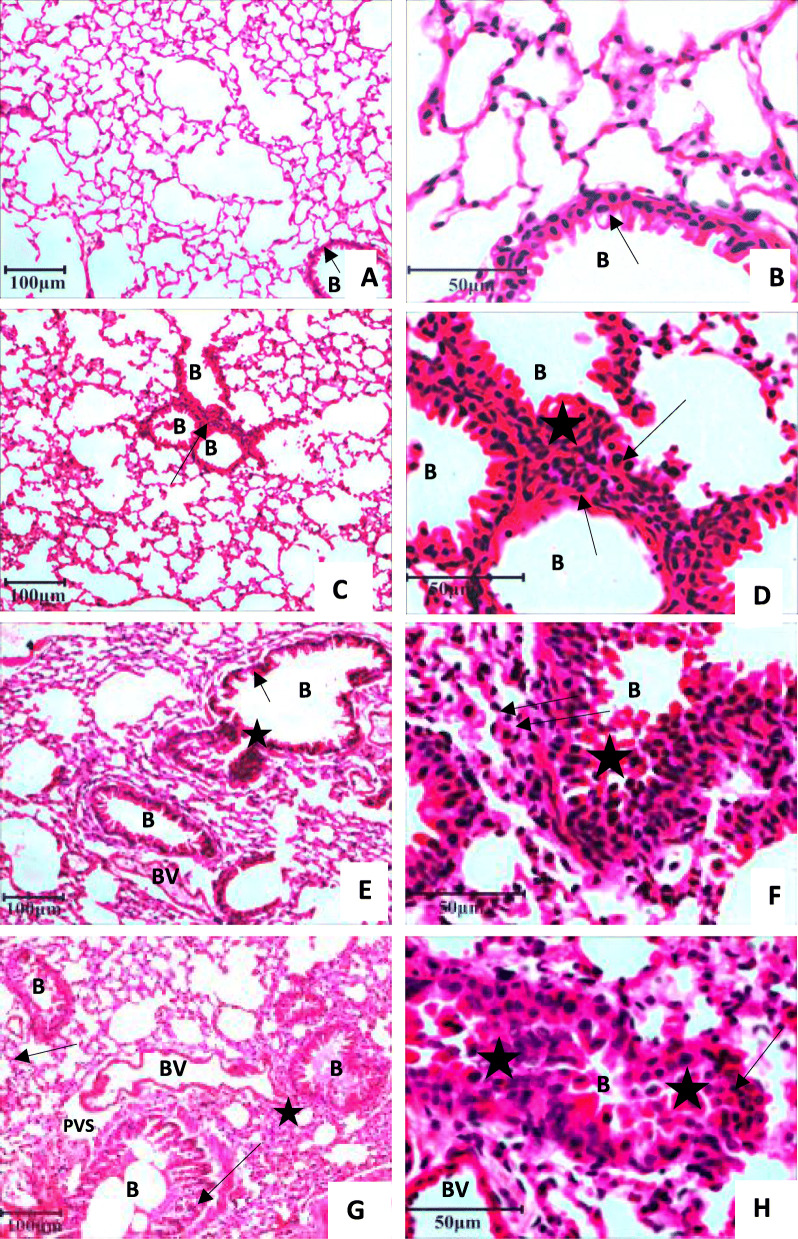
Haematoxylin and Eosin staining of Paraffin sections of lung showing normal histoarchitecture (**A**, **B**); peribronchial mononuclear infiltrating cells (arrow) and infiltration of inflammatory cells around alveolar septa (star) following treatment with LPS (**C**, **D**), ethion alone (**E**, **F**), or in combination with LPS (**G**, **H**). B: Bronchiole; PVS: Perivascular space; BV: Blood vessel; Original magnification A, C, E, G: 10 X; B, D, F, H: 40 X

**Fig. 4:**
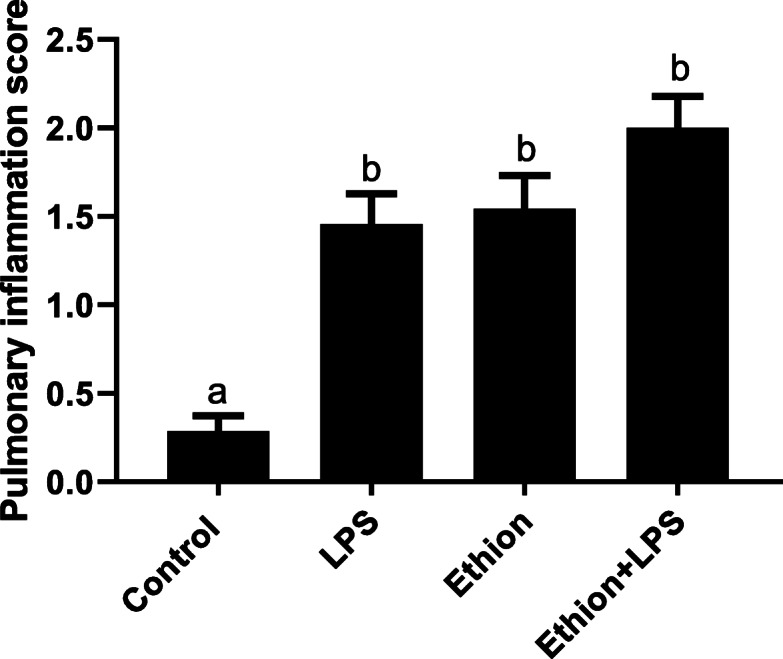
Histopathological changes in lung expressed as pulmonary inflammation score after single dietary exposure to ethion for 24 h alone and with LPS. The results of five samples from each group are expressed as mean ± SE. Different superscript letters between two levels of an effect indicate significant difference (*p* < 0.05)

The paraffin lung section from control group exhibited normal histoarchitecure of the alveolar septa and airways epithelium (Fig. 3A, B). LPS and ethion exposure alone increased peribronchial infiltration along with infiltration of the mononuclear cells in the alveoli (Fig. 3C-F). The damage following co-exposure to ethion and LPS together was characterized by peribronchial and perivascular infilteration, sloughing of airways epithelium and expanded perivascular space (Fig. 3G, H). Semiquantitative histology revealed that treatment with LPS or/and ethion significantly increased (*p* < 0.05) pulmonary inflammation score compared to control (Fig. 4).

### mRNA and protein expression

#### Toll-like receptor 4

**Fig. 5:**
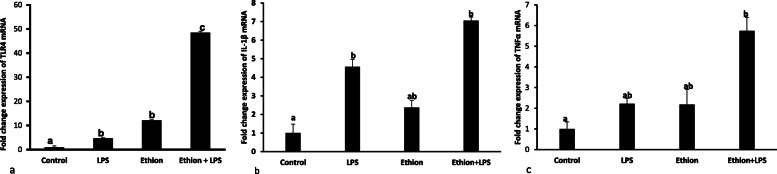
**a** Fold change expression of TLR4 **b** IL-1β and **c** TNFα mRNA after single ethion exposure for 24 h alone or along with LPS challenge. **a**,**b**,**c**:No common superscript between two levels of an effect indicates significant difference (*p* < 0.05)

Ethion with or without LPS significantly increased the mRNA expression of TLR4 compared to control. Further, combination of ethion and LPS caused significant increase in TLR4 mRNA compared to ethion or LPS group. There was 4.68, 12.18 and 48.53 fold increase in the mRNA expression of TLR4 following exposure to LPS, ethion and combination of ethion and LPS, respectively (Fig. 5a).

**Fig. 6:**
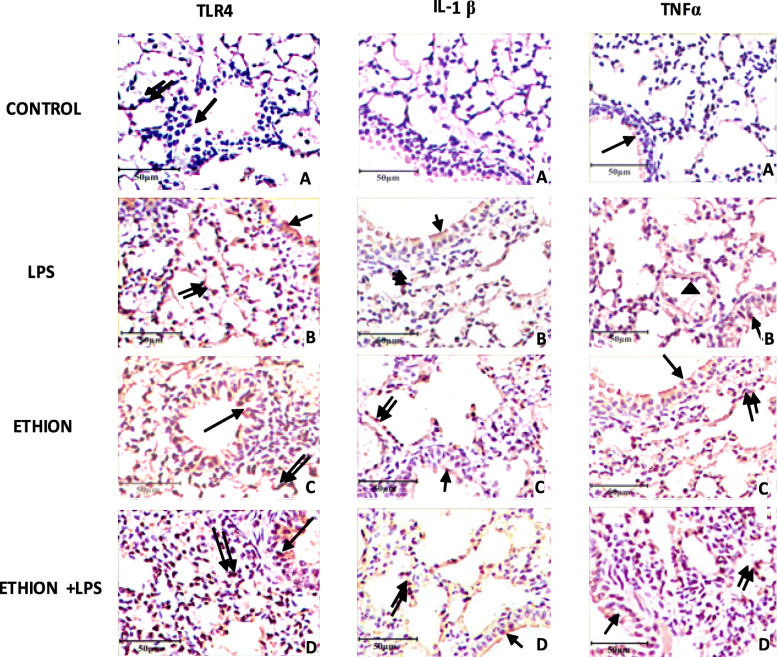
Lung section showing immunopositive reaction in alveolar cells (single arrow), septal cells (double arrow) and endothelial cells of large blood vessel (arrow head). Representative images of TLR4, IL-1β, and TNF-α immunopositive cells after exposure to single ethion exposure and LPS. Original magnification: × 40

Exclusion of primary antibody or both primary and secondary antibodies resulted in lack of staining in the tissue sections (data not shown). The airways epithelial and alveolar septal cells in the lungs of control animals showed a weak TLR4 immunoreactivity (Fig. 6 A). LPS and ethion alone or in combination resulted immunopositive TLR4 reactivity in septal and airways epithelial cells (Fig. 6B-D). TLR4 reactivity was localized in cytoplasm of airway epithelial cells.

#### Interleukin-1β

LPS resulted 4.56 fold increase (*p* < 0.05) in IL-1β mRNA expression compared to control group (Fig. 5b). Ethion did not alter the mRNA expression of IL-1β. Further, ethion in combination with LPS caused 7.05 folds increase (*p* < 0.05) in IL-1β mRNA expression compared to control group but did not vary from individual LPS or ethion group.

There were few immunopositive cells for IL-1β in normal healthy group (Fig. 6A). LPS and ethion alone or together showed strong immunopositive reaction for IL-1β in airways epithelium and alveolar septa (Fig. 6B-D). IL-1β immunopositive reactivity was localized in nuclei and cytoplasm of alveolar macrophages.

#### Tumor necrosis factor-α

As shown in Figure 5 c, Ethion or LPS alone did not show any significant increase in TNF α mRNA expression compared to control. Further, combination of ethion and LPS increased (*p* < 0.05) the TNFα mRNA expression (5.57 folds) compared to control (Fig. 5c).

Control group showed weak TNF-α positive reaction. LPS treated mice lungs showed TNF-α immunopositive reaction (Fig. 6B). The reaction was positive in bronchial epithelial cells, septal cells and endothelial cells of large blood vessel. Single dietary ethion exposure alone or together with LPS also showed TNF-α immunopositive reaction in bronchial epithelial and septal cells (Fig. 6 C-D). TNF-α positive staining was observed in alveolar septal cells, endothelial cells and epithelial cells of bronchiole along with infiltrating cells in lungs of mice.

### Single cell gel electrophoresis

**Fig. 7:**
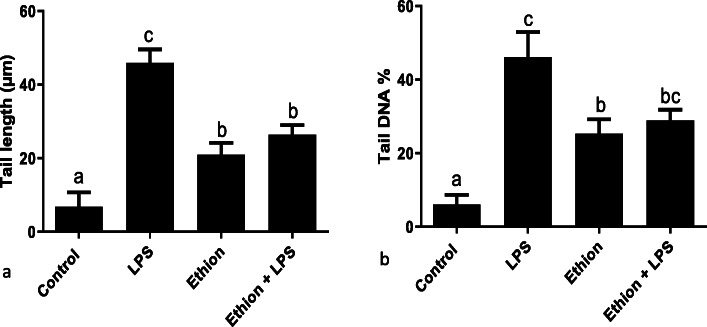
**a** Tail length (μm) and **b** tail DNA% after single ethion exposure with LPS. a,b,c,d: no common superscript between two levels of an effect indicates significant difference (*p* < 0.05) among groups

The healthy cells showed intact nucleus without any comet, however damaged cells had a comet. LPS or ethion exposure resulted in significant increase (*p* < 0.05) in tail length and tail DNA% compared to control (Fig. 7). Further, LPS showed more significant increase (*p* < 0.05) in both parameters compared to ethion. Ethion combined with LPS did not show any significant differences compared to ethion alone. There was a significant strong correlation (*r* = 0.99; *p* = 0.010) between tail length and tail DNA%.

## Discussion

The present study evaluated the pulmonary effects of oral administration of ethion alone or in combination with LPS in a mouse model. We present the first data suggesting that single low dose dietary exposure to ethion causes lung damage and alter the pulmonary expression of TLR4 mRNA.

Single exposure to ethion at 8 mg kg^− 1^ for 24 h resulted decrease in TLC along with lymphocytopenia indicating direct or indirect damage to lymphocytes. Coumaphos induces significant decrease in leukocyte count, absolute lymphocyte, erythrocyte and platelet counts in healthy male steers [27]. There was significant neutrophilia after ethion exposure and neutrophilia with lymphocytopenia is associated with organophosphate toxicity in rats [28] and with lambda-cyhalothrin, a pyrethroid insecticide, in female rabbits [29].

BAL fluid analysis forms an indispensable part to study lung inflammation. LPS exposure significantly increased TLC and neutrophil % of BAL fluid which is a characteristic of lung inflammation. Single dietary ethion exposure significantly increased the neutrophil % in BAL fluid compared to control group as reported earlier following exposure to chlorpyriphos [15] and carbaryl [30]. Infiltration of activated neutrophils into the lung and BAL fluid is an important component of the inflammatory response during acute lung injury [31]. Ethion followed by LPS increased (*p* < 0.05) TLC and neutrophil % of BAL fluid compared to LPS alone suggesting that pre-treatment with ethion alter response to LPS.

The histopathological observations revealed significantly increased pulmonary inflammation score following ethion exposure which was apparently consistent with the BAL cytology. Ethion exposure combined with LPS showed sloughing of airways epithelium, expanded perivascular space, peribronchial and perivascular infilteration of cells suggesting lung injury. Acute exposure to diazinon [32] in guinea pig and phosgene [33] in mice causes lung damage. The pesticide induced pulmonary alterations could be attributed to the decrease in the antioxidant status [34]. The histopathological observations along with BAL fluid analysis indicate ethion induced lung damage.

TLR4 gets activated upon recognization of LPS and activated TLR4 results in production of proinflammatory mediators like IL-1β and TNF-α in airways and epithelial cells via NF-kB pathway [9]. TLR4 also plays an important role in acute lung injury induced by herbicide, paraquat [6]. Single ethion exposure resulted in significant increase in the expression of TLR4 mRNA and TLR4 immunopositive reactivity. Pretreatment with ethion also significantly enhanced the LPS induced TLR4 pulmonary expression suggesting synergistic effect of ethion and LPS to activate TLR4. The synergistic interaction of LPS and pesticide affects the cytokine expression [13], apoptosis [14] and lung immunity [15]. The data shows that stress on lung is worsened when ethion is combined with LPS.

Activated TLR4 increases the levels of IL-1β via an Ice Protease-Activating Factor (IPAF) dependent and caspase independent pathway [10]. IL-1β is one of the most central cytokines involved in the acute lung inflammation [35]. It induces the expression of abundant downstream signaling effector molecules of acute phase inflammation [36]. Ethion exposure did not alter the pulmonary expression of IL-1β mRNA whereas; IL-1β immunopositive reaction in alveolar septal cells and airways epithelium was observed. There may be a possibility that we might have overlooked the time point when IL-1β mRNA was altered. However, ethion in combination with LPS significantly increased the IL-1β mRNA expression. Low levels exposures to insecticide acephate enhance responses to LPS induced pro-inflammatory cytokines IL-1β, TNFα, and IFN-γ in rats [37].

Activation of epithelial proinflammatory signaling cascades is mediated by TNF-α which regulates broad spectrum of responses to stress and injury [11]. Individual treatment with ethion or LPS did not show any significant increase in TNFα mRNA expression however, the most pronounced increase in TNFα mRNA was seen only when mice were treated with both ethion and LPS suggesting synergistic response of ethion and LPS. Interestingly, ethion or LPS alone or in combination showed TNF-α immunopositive reaction. Similarly, parathion treatment of alveolar macrophages did not significantly increase TNF-α mRNA but significantly increase TNF-α protein release in guinea pigs [38]. TNF-α facilitate migration of neutrophils into inflamed lungs, hence, the higher levels of lung inflammation observed in mice exposed to ethion and LPS may be due to increased expression of cytokines such as TNF-α.

The assessment of genotoxic potential of a pesticide is of prime importance in the field of genetic toxicology. Tail length and tail DNA% are very reliable parameters to predict DNA damage [39]. The presence of comet and significant increase in tail parameters following individual exposure to ethion or LPS was observed. Ethion is known to induce genotoxicity in chicks [40] and *Anopheles culicifacies* [41]. Similarly, LPS induces indirect DNA damage in peripheral blood mononuclear cells of human and mice which might be due to induction of oxidative stress [42]. LPS activates macrophage and production of nitrite and nitrating agent that damages the cell membrane resulting DNA damage and cell death [43]. The data taken together suggest single dietary exposure to ethion at 8 mg kg^− 1^ has the potential to cause genotoxicity.

The present study did not validate the mechanism(s) involved in production of inflammatory mediators after ethion exposure. Secondly, the acute change within 24 h could be affected by several other factors and it could be transient change hence data beyond 24 h exposure need to be compared. However, the enhanced level of TLR4, IL-1β and TNF-α mRNA expression after ethion is combined with LPS as observed in the present and earlier studies [2, 7] depicts that these could serve as potential markers in ethion induced lung injury and could also serve as targets for therapy research. The present study encourages further experimentation on the human pulmonary cell lines. Effective therapies can be developed in future to mitigate pulmonary effects induced by ethion exposure based on knowledge of mechanism(s) and mediators involved in ethion induced lung injury.

## Conclusions

We conclude that single dietary ethion exposure at 8 mg kg^− 1^ cause lung inflammation, alter lung histology and pulmonary expression of TLR4 mRNA. Furthermore, pre-treatment with ethion produces synergistic response to LPS induced expression of TLR4 mRNA. However, further comprehensive studies are needed for understanding the role of the molecular pathway(s) dysregulated during ethion induced lung damage and to identify other vulnerable target organs.

## Data Availability

The data supporting this study are available on request from the corresponding author.
